# Near patient chlamydia and gonorrhoea screening and treatment in further education/technical colleges: a cost analysis of the ‘Test n Treat’ feasibility trial

**DOI:** 10.1186/s12913-020-5062-5

**Published:** 2020-04-16

**Authors:** Sarah Kerry-Barnard, Susie Huntington, Charlotte Fleming, Fiona Reid, S. Tariq Sadiq, Vari M. Drennan, Elisabeth Adams, Pippa Oakeshott

**Affiliations:** 1 Population Health Research Institute, St George’s, University of London, London, SW17 ORE UK; 2 Aquarius Population Health Limited, Unit 29, Tileyard Studios, Tileyard Rd, London, N7 9AH UK; 3 School of Population Health and Environmental Sciences, King’s College London, London, SE1 1UL UK; 4 Institute for Infection and Immunity, St George’s, University of London, London, SW17 ORE UK; 5 Centre for Health and Social Care Research, Kingston University and St George’s University of London, London, SW17 ORE UK

**Keywords:** Test n treat, Chlamydia, Gonorrhoea, Cost analysis, Genitourinary infection, Health services

## Abstract

**Background:**

Community-based screening may be one solution to increase testing and treatment of sexually transmitted infections in sexually active teenagers, but there are few data on the practicalities and cost of running such a service. We estimate the cost of running a ‘Test n Treat’ service providing rapid chlamydia (CT) and gonorrhoea (NG) testing and same day on-site CT treatment in technical colleges.

**Methods:**

Process data from a 2016/17 cluster randomised feasibility trial were used to estimate total costs and service uptake. Pathway mapping was used to model different uptake scenarios. Participants, from six London colleges, provided self-taken genitourinary samples in the nearest toilet. Included in the study were 509 sexually active students (mean 85/college): median age 17.9 years, 49% male, 50% black ethnicity, with a baseline CT and NG prevalence of 6 and 0.5%, respectively. All participants received information about CT and NG infections at recruitment. When the Test n Treat team visited, participants were texted/emailed invitations to attend for confidential testing. Three colleges were randomly allocated the intervention, to host (non-incentivised) Test n Treat one and four months after baseline. All six colleges hosted follow-up Test n Treat seven months after baseline when students received a £10 incentive (to participate).

**Results:**

The mean non-incentivised daily uptake per college was 5 students (range 1 to 17), which cost £237 (range £1082 to £88) per student screened, and £4657 (range £21,281 to £1723) per CT infection detected, or £13,970 (range £63,842 to £5169) per NG infection detected.

The mean incentivised daily uptake was 19 students which cost £91 per student screened, and £1408/CT infection or £7042/NG infection detected.

If daily capacity for screening were achieved (49 students/day), costs including incentives would be £47 per person screened and £925/CT infection or £2774/NG infection detected.

**Conclusions:**

Delivering non-incentivised Test n Treat in technical colleges is more expensive per person screened than CT and NG screening in clinics. Targeting areas with high infection rates, combined with high, incentivised uptake could make costs comparable.

**Trial registration:**

ISRCTN58038795, Assigned August 2016, registered prospectively.

## Background

*Chlamydia trachomatis* (CT) and *Neisseria gonorrhoeae* (NG) are bacterial sexually transmitted infections (STI), responsible for almost half of STI diagnoses in England and 62% in people aged 15–24 years [1]. However, uptake of testing in many countries is too low to reduce infection rates, and there may be delays in obtaining treatment. As both infections can be symptomless, they can go undetected leading to problems such as pelvic inflammatory disease, epididymitis, infertility and adverse birth outcomes [2, 3]. Although NG is less common than CT, it is a potentially more serious STI over which there are concerns about antibiotic resistance [4]. Therefore, in this study, participants diagnosed with NG were referred to specialist clinics for further management [5, 6].

Self-collected vaginal swabs (for females) and first void urine samples (for males) are ideal sample types for CT and NG testing. Combining this with portable point of care (POC) rapid test platforms gives potential to test for these infections in a variety of community settings, allowing people to receive results on the same day as testing. Using POC CT/NG tests in high prevalence settings may help reduce the burden of disease by making testing more convenient and providing results faster, thereby reducing the time to treatment [7, 8]. However, data on the costs of providing community-based services are limited. Previous cost analyses have primarily focused on using POC CT/NG tests in clinical settings and/or based on modelled data [9–11]. There is an urgent need for real life data to explore the economics and practicalities of screening and treatment of STIs in the community.

We used field data from the ‘Test n Treat’ (TnT) feasibility trial of screening for CT/NG in further education/technical colleges [5, 6]. (Further education colleges offer both academic and practical courses such as plumbing and hairdressing and take many students from socio-economically deprived backgrounds.) The feasibility trial aimed to measure uptake and acceptability of on-site rapid STI testing and treatment to students, and recruitment and follow up rates. In the current economic analysis, we estimated the cost per person screened and the cost per CT/NG infection detected for:

- Non-incentivised testing
- Incentivised testing
- Maximum possible uptake (using incentives)

## Methods

### Aims

To estimate the costs of running a ‘Test n Treat’ service providing rapid CT and NG testing and same day on-site CT treatment in technical colleges and to estimate the cost per person screened and per CT/NG infection detected for: non-incentivised/standard testing; incentivised testing-when participants were given £10 to be tested; and with maximum possible uptake (using incentives).

### Intervention

The TnT cluster randomised feasibility trial recruited sexually active students attending six technical colleges in South London. ‘Test n Treat’ refers to testing students for CT and NG on site at their college, giving them a same day result, and offering same day on-site treatment from a health adviser for students with a positive CT test. (As mentioned earlier, students with NG were referred to a sexual health clinic for specialist management.)

**Fig. 1:**
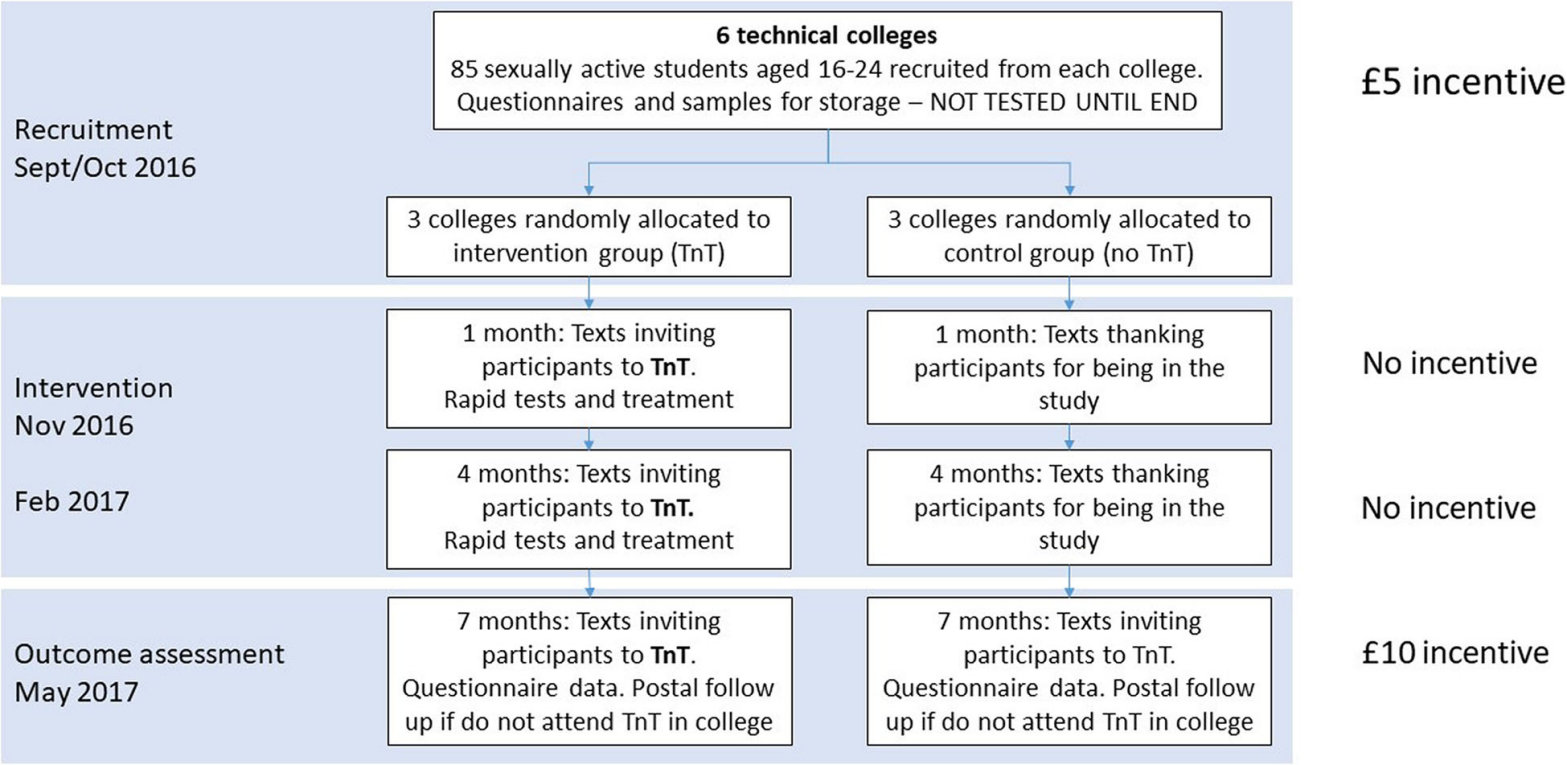
TnT flow chart

The protocol and main results of the TnT trial are available elsewhere [5, 6]. In summary, 509 sexually active ethnically diverse students aged 16–24 years were recruited from communal areas in six technical colleges in South London in October 2016 (Fig. 1). Participants completed questionnaires on sexual lifestyle and provided self-taken genitourinary samples. We provided information about the risks of CT/NG and explained that as these baseline samples would not be tested for 7 months all participants should seek STI testing at a sexual health clinic or from their family doctor independently of the trial.

Three of the colleges were randomly assigned to receive TnT visits one and four months after recruitment. Students previously recruited at these sites were texted/emailed invitations to come to a classroom for confidential testing and same day on-site treatment. As previously, they were asked to provide self-taken urine samples (males) or vaginal swabs (females) in the nearest toilet. (In women, vaginal swabs are more reliable than urines for detection of CT.) Samples were tested immediately in a “pop-up” laboratory at the college using the GeneXpert® CT/NG test (Cepheid, Sunnyvale, CA, USA) yielding a result in 90 min [12]. The GeneXpert4/4 s machine - a portable unit weighing 10 kg - contains four modules which can be used asynchronously to test four separate samples. Three machines were used at each TnT visit allowing 12 samples to be tested simultaneously.

Negative results were texted to participants (in a median time of 2.1 h after providing a sample). The research team’s nurse health adviser telephoned participants with positive results and met them in another private room in college (same day whenever possible) for confidential treatment for CT, partner notification and/or referral (for NG management). Students with CT who did not attend for treatment in college, and those with dual CT/NG infection, were referred to a sexual health clinic for further management.

In the three control colleges, students received text messages 1 month and 4 months after recruitment thanking them for their participation. For the outcome assessment exploring change in CT prevalence, the TnT service was offered in all six colleges at seven months - when students received a £10 incentive for providing a follow up sample (and questionnaire). The incentive was suggested by our PPI group and intended to maximise follow up.

### Calculating costs and consumables

There were two types of costs involved in providing the TnT service: the fixed or “daily” costs, i.e. costs incurred irrespective of the number of people screened; and the per person costs, i.e. the variable costs dependent on the number of people screened. The components of delivering TnT included the cost of two healthcare assistants organising CT/NG screening, and a health advisor providing treatment where CT was diagnosed, plus the cost of travel, all equipment and consumables (Additional file 1: Supplementary Table 1). There was no charge from the participating colleges for use of their rooms. (This is usual practice for visiting services which are provided free and may benefit students.) Costs reflect 2018 prices and are presented in British pounds. Currency conversion rates are taken from 1st June 2018, and inflation rates are taken from mid-year [13, 14].

### Uptake scenarios used to calculate screening costs

Variation in testing uptake impacts the per-person cost of providing the service. As such, three different scenarios are reported based on uptake:

1. The average (a), minimum (b), maximum (c), half the average (d) and double the average (e), number of students who used the non-incentivised service.
2. The average number of students who used the incentivised service (when students received £10 for participation).
3. The maximum number of students who could use the service if it were run at full capacity.

Scenario 1 was calculated using the TnT data from the one and four-month visits at the three intervention colleges, which were two days each (12 days in total). Scenario 2 was calculated using the incentivised two-day TnT seven months follow up at all six colleges (12 days in total). For Scenario 3, trial design meant using a pragmatic simulation model to show the maximum number of students who could be tested in a day with two staff, three machines and incentives. The reason for doing this was that the trial design meant we only invited the 85 students already recruited at each college to TnT. If TnT were rolled out to all colleges in real life, all students at the college (range 500–3000 per college) would be invited for testing. With incentives it is possible that maximum daily capacity would be achieved. The maximum capacity of the service (Scenario 3) was calculated by creating a simulation model in Python (Python Software Foundation. Python Language Reference, Version 2.7) [15]. Details are outlined in Additional file 2; Additional file 5: Supplementary Figure 1, and Additional file 6: Supplementary Figure 2.

### Testing and treatment pathway

In order to calculate total costs, a testing and treatment pathway was mapped containing the different cost contributions and uncertainties. For non-incentivised and incentivised scenarios, the following pathway parameters were calculated based on the field data.

- Ratio of male to female participants (because the samples from males and females took different times to process)
- Proportion of tests that failed on first run (for example, because samples did not contain human DNA) and were repeated
- Proportion of tests that failed because the sample was inadequate or missing, and therefore the student was asked to provide another sample
- Proportion of repeat samples provided/not provided
- CT positivity rate
- NG positivity rate

### Primary outcomes

Three primary outcomes, based on outcomes used by the National Chlamydia Screening Programme (NCSP) [16], were estimated for each uptake scenario:

1. The cost per student screened for CT/NG
2. The cost per CT infection detected
3. The cost per NG infection detected

For each uptake scenario, the cost per student screened was calculated as: (fixed daily cost + [per screen cost x number of screens])/number of screens. The cost per CT or NG infection detected was calculated as: cost per student screened x number of screens needed to diagnose one infection (i.e. 100/% prevalence).

### Further analyses

Each college was visited for two consecutive days and there were typically fewer tests performed on the second day. Where the CT/NG testing platforms were at full capacity during college hours or repeat testing was needed, tests were performed in the evening after students had left college, or the following day. For each scenario, the number of test results that were not given on the same day as the sample was provided is reported, as well as the number of students who could not be given a test result because they did not give a valid sample. The model parameters for estimating maximum capacity were based on averages per TnT session: 49:51 male; 5% repeated samples; 20% of failed tests need to be resampled of whom 25% provide a second sample; 5% CT positivity rate.

### Patient and public involvement

Focus groups were used to inform the study design of the feasibility trial including incentives. The steering committee included four student representatives.

## Results

### CT/NG screening uptake

In total, 291 samples were tested from 254 students: 59 tests when no incentive was given, (at the two intervention visits) and 232 when £10 incentives were given (at the follow-up visit). Half the students who used the service were female (51%, 130/254), and the median age was 17.9 years.

There was an average of 85 participants recruited per college. Students from college A provided 26 samples during the non-incentivised TnT interventions and 37 during the incentivised follow-up. College B and college C provided 14 and 31 samples, and 19 and 33 samples, respectively. The three control colleges D, E and F provided 48, 43 and 43 samples at the incentivised follow up. Ten samples (3%) required repeat testing. Of these, four had to be resampled as they were invalid. Invalid samples (with no human DNA) only occurred during the incentivised follow-up. Only one student provided a second valid sample, and so three people could not be given a result. Overall, 5.8% (17/291) of samples tested positive for CT and 1.4% (4/291) for NG.

Each of the intervention colleges A-C was visited on two consecutive days at 1 and 4 months. The mean number of students tested each day per college at the non-incentivised service was five, the highest uptake in one day was 17. There were two days when only one student used the service. By comparison, the average daily number of students who used the incentivised service per college (in all colleges A-F at 7 months follow-up) was 19 (232 over 12 days) (Table 1).

**Table 1:** Screening costs of providing TnT in technical colleges

Uptake scenario [Students screened per day, per college]		Cost per student	Cost per CT infection detected	Cost per NG infection detected
1a. Average uptake non-incentivised	[*n* = 5]	£236.77	£4656.55	£13,969.66
1b. Lowest uptake non-incentivised	[*n* = 1]	£1082.06	£21,280.52	£63,841.56
1c. Highest uptake non-incentivised	[*n* = 17]	£87.61	£1722.91	£5168.73
1d. Half the average uptake non-incentivised	[*n* = 2.5]	£448.10	£8812.54	£26,437.63
1e. Double the average uptake non-incentivised	[*n* = 10]	£131.11	£2578.56	£7735.67
2. Average incentivised uptake*	[*n* = 19]	£91.06	£1408.44	£7042.22
3. Maximum incentivised uptake*	[*n* = 49]	£47.02	£924.64	£2773.92

Footnotes:

Scenario 1a: The average number of students who used the non-incentivised service.

Scenario 1b: The minimum number of students who used the non-incentivised service.

Scenario 1c: The maximum number of students who used the non-incentivised service.

Scenario 1d: Half the average update for the non-incentivised service.

Scenario 1e: Double the average update for the non-incentivised service.

Scenario 2: The average number of students who used the incentivised service.

Scenario 3: The maximum number of students who could use the service if it was run at full capacity using three 4-unit machines.

*Costs for scenarios 2 and 3 include £10 per student incentive

Data for 27 tests (9% of total) run after 5 pm on the first of a two-day TnT visit are included in the calculations

### Pathway timing

**Fig. 2:**
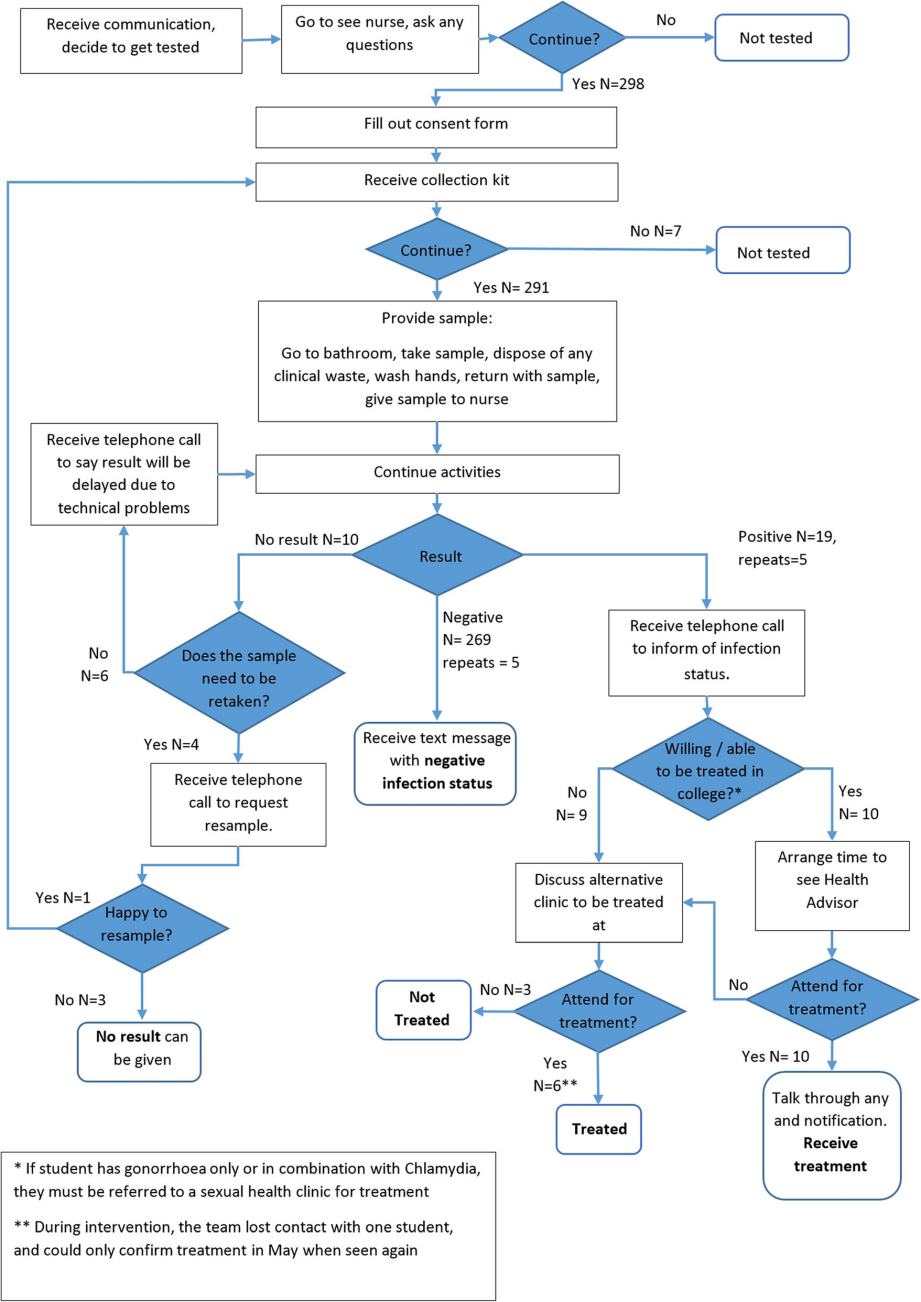
Student pathway flowchart showing possible testing scenarios with field data

The median time for sampling, testing and receiving the results of the CT/NG test was 129 min, including the time needed to repeat test 10 samples. For a negative result, the median time was 128 min and for a positive result it was 160 min. Each step involved in sampling, testing and reporting results to students are presented in Fig. 2, and the associated times are presented in Additional file 7: Supplementary Table 3. Practical notes from delivering the TnT service are presented in Additional file 4 and the steps required to deliver TnT as a service and for research are presented in Additional file 3: Supplementary Table 2.

### Estimated costs

The fixed daily cost per college visit was £1056.61. This comprised of staff costs (£753.60); courier services delivering three machines (£110.40), diagnostic equipment hire (£184.20) and consumables (£8.41) (Supplementary Table 1). Each screen performed cost an additional £25.45, accounting for the small number of repeat tests and the additional £2 for the cost of antibiotics to treat those diagnosed with CT.

### Estimating maximum capacity

**Fig. 3:**
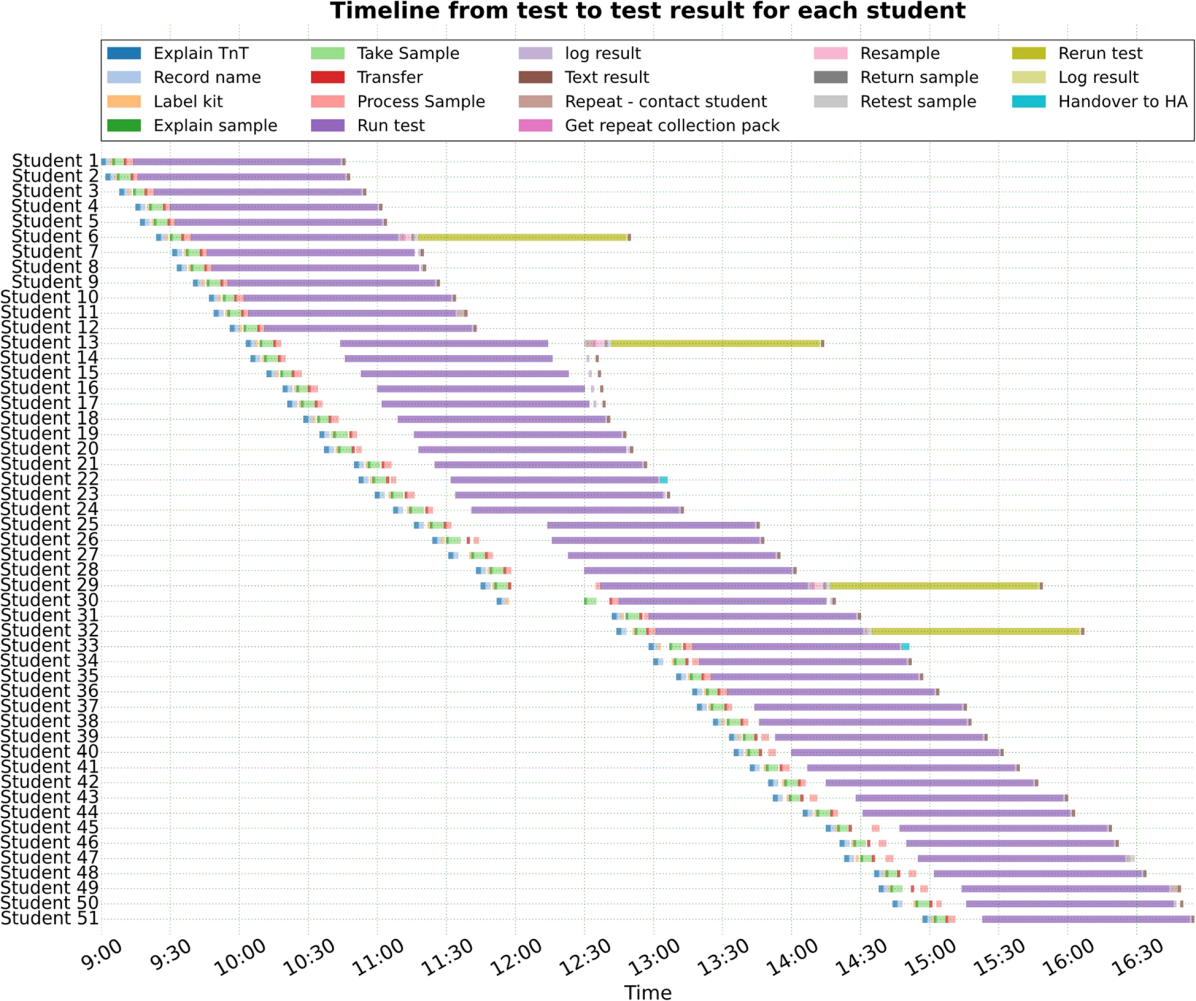
Gantt chart showing each student’s pathway in one simulated day of TnT

The model found that the maximum number of students who could attend the service if it was run at full capacity using three 4-unit machines was 49 per day. Figure 3 shows the timeline for each student screened in scenario 3.

### Footnotes

The simulation was based on pathway data from 24 days of providing the TnT service.

In scenario 3, there was insufficient remaining time to test all the samples that required repeat testing (1% of total).

‘Handover to HA’ refers to the health advisor being given the details of students with a positive result so that they could contact them to provide treatment and arrange partner notification.

‘Text result’ is for negative results only.

### Screening costs

The cost per student screened and per CT/NG infection detected for the different uptake scenarios (accounting for the fixed daily costs and the per screen costs) are presented in Table 1.

For the non-incentivised service, where CT prevalence was 5.1% (3/59) and NG prevalence 1.7% (1/59), the mean cost per student screened was £237 and the cost per CT/NG infection detected was £4657/£13,970 respectively.

For the incentivised service, where CT prevalence was 6.5% (15/232) and NG prevalence 1.3% (3/232), the cost per student screened was £91 and the cost per CT/NG infection detected was £1408/£7042 respectively.

Just as higher uptake of the service reduced the cost per screen, in settings with higher CT prevalence the cost per CT infection detected would be lower. For example, if the prevalence of CT was 8%, the cost per CT infection detected would be £2960/£1138/£588 for non-incentivised, incentivised and maximum incentivised uptake (i.e. scenarios 1a, 2 and 3) respectively. At 10% CT prevalence, the cost per CT infection detected would be £2368/£911/£470 for non-incentivised, incentivised and maximum incentivised uptake respectively.

## Discussion

### Principal findings

Using the non-incentivised TnT screening service piloted in FE colleges, the estimated cost per student screened for CT/NG, was £237 for the average daily uptake of the service (5 students/day), or £88 per student screened at times of highest use of the service. With highest use (17 students/day) the cost per CT/NG infection detected was £1723/£5169 respectively. Students were notified of their results quickly: average time to notification was just over two hours. There was a high fixed cost (over £1000 per day) for providing the service-mainly staff costs. This means there is a high cost per student screened when uptake of the service is low. However, incentives may be cost effective for case detection. Our model suggested that if using incentives achieved maximum capacity (49 students/day), the cost per student screened and per CT infection detected would be much lower: £47 and £925 respectively.

### Strengths and weaknesses

This is the first UK study to use “real-life” field data as a basis for cost analysis of providing POC CT/NG services outside of clinical settings, and the first to evaluate the use of rapid tests and treatment in the community. The list of resources used in the TnT study are presented and could be used as a “how-to” guide for sexual health services wanting to provide this type of service within colleges or other community-based settings. Incentivisation could therefore increase uptake and reduce the per-person and per-infection screening costs as average attendance was much higher in the incentivised scenario. TnT may be reaching students who would not otherwise get tested. Although at recruitment we advised all participants to get tested outside the trial, only 27% of responders reported doing this.

This type of service could reach groups such as sexually active adolescents who might otherwise be reluctant to access community sexual health services or to have postal CT tests sent to their home. The majority of students who were screened as part of TnT did not have a CT test outside of TnT [6], despite being advised to do this at recruitment. Most participants (90%) were teenagers, and almost half of those tested were male, in contrast to the NCSP which screens a much higher proportion of females, (despite being an opportunistic screening programme for any gender) [16]. Males may be happier to engage with community-based screening than screening in a clinical setting [17, 18].

The main weakness is that testing uptake may have been limited by the study design – only students recruited and consented into the study were eligible to use the service. During our earlier one-day pilot of TnT when any sexually active student could take part, attendance was considerably higher (34 per day) [19]. Using the same cost data, the cost per screen in the pilot was £56.53 and the cost per CT infection detected, £640.66 (as CT prevalence was 11% [3/34]). Secondly, as all students were already part of the TnT study, they already had some knowledge of testing from providing samples at recruitment and were contacted directly to participate. This may have increased participation. Selection bias may have occurred both at recruitment and during testing. However, the prevalence of CT infection in incentivised and non-incentivised TnT was similar (6 and 5% respectively), suggesting that providing incentives did not increase levels of testing disproportionally in low-risk groups - as has been the concern with other screening strategies [16].

Another limitation is that although most test times were documented, some were estimates. Real times will vary according to students’ familiarity with providing samples and staff skills, as well as locational variables such as the distance to the nearest bathroom and the distance between the lab and the meeting space. These estimates were however based on experience in the field and, where possible, repeated measurements were taken. The colleges did not charge for use of rooms, but these costs might need to be added for screening in other settings. Finally, findings may not be widely applicable. This study focused on six technical colleges in south London, an area which has good transport links and where there is access to multiple NHS sexual health services. Costs, particularly costs associated with travel and venue hire, may be higher in other settings. Uptake of services may also be different in other settings or among older or less ethnically diverse groups, something which would impact average costs.

The small number of students screened per day and the high fixed cost of providing the service means that the per student cost was very sensitive to changes in the number of students screened per day. Compared to average update for the non-incentivised service, if twice as many students were screened, the cost per student decreased considerably (from £236.77 for 5 screens/day to £131.11 for 10/day) and if half as many students were screened it increased considerably (to £448.10 for 2.5 screens/day).

### Comparison with other studies

The estimated costs under the average non-incentivised conditions in this feasibility study (£237 per student screened) were considerably higher than the London Integrated Sexual Health Tariff (ISHT) for a CT/NG test used in numerous clinical settings within the NHS (National Health Service), which was £45 per attendance in 2017/2018 [20]. They are also higher than the estimated cost of opportunistic CT screening in the UK in 2011 – estimated at £61 per CT screening episode for 2018 (inflated from £51 for NCSP 2011 data) [16], and in Ireland estimated at £23 per offer (in 2018, inflated and converted from €26 in 2008) [21, 22]. If demand for the TnT service was very high and the service was run at full capacity, the cost per student tested would be £47. This cost is closer to the ISHT and NCSP screening costs. In addition, if demand were that high, it is likely the service would be extended over a longer period of time to meet the demand which would impact costs further.

There have been cost analyses of non-clinic-based screening in other countries. In “Stamp out Chlamydia”, an Australian community screening study, the non-incentivised cost per person screen was £128 (inflated and converted from 2007 data) [17]. A more recent study of routine repeat screening in the Netherlands reported screening costs of £100 per-screen (inflated and converted from 2014 data) [22]. A community CT screening study in England aimed at men attending sports clubs estimated that costs ranged from £92 to £100 per screen (inflated from 2013 estimates) with no CT infections detected [23].

The high prevalence of CT in the participating FE colleges (5.1% non-incentivised and 6.5% when incentivised) resulted in lower costs per infection detected compared to similar screening studies. The estimated cost per CT infection diagnosed was £4657 when using data for non-incentivised average daily uptake of the service and £1723 when using data for highest non-incentivised uptake. This is comparable with both the “Stamp out Chlamydia” study and the Netherlands based routine screening study which reported costs of £5395 and £5053 per CT infection detected respectively (inflated to 2018 costs) [17, 22]. The TnT model had advantages over these testing strategies in that it also screened for NG and provided same day results and treatment for CT. Finally, the estimated incentivised costs in the “Stamp out chlamydia” study were £20 per-screen and £2378 per CT infection diagnosed compared to £91 per-screen and £1408 per CT infection detected for TnT. In both studies when screening was incentivised, the per-screen and per-CT infection diagnosed costs were less while the rate of CT detection was relatively unchanged.

## Conclusion

Although resource intensive, with sufficient uptake and high rates of STIs, delivering the TnT service in non-clinical settings may cost a similar amount to CT/NG testing in clinics. The higher screening cost could be justified if people using the service were unlikely to use other less costly services such as postal screening or attending clinics. It could also be appropriate in settings where community health services are sparse or difficult to access or where other types of screening for CT/NG are not available or well accepted.

Higher uptake of the service would considerably reduce the cost per screen. Our study suggests incentivising testing could help increase uptake without reducing positivity rates. As shown in the process evaluation, incentives might also reduce stigma as people can imply that they are just getting tested for the money. However, we found this needs to be carefully managed to avoid abuse (such as impersonation or providing invalid samples). Finally, our participants’ lack of awareness of STIs and the need for testing highlight the need for better sex education for young people and for making regular STI checks routine.

## Supplementary information

**Additional file 1: Supplementary Table 1**. Costs and consumables required to deliver the Test n Treat service. The costs incurred throughout the study are listed in Supplementary Table 1, along with a description of the cost, purchase unit, quantity, type of cost and source of information are also included.

**Additional file 2.** Calculating the maximum capacity of the TnT service (Scenario 3)

**Additional file 3 Supplementary Table 2.** Processes required to deliver Test n Treat as research and as a service. All the procedures performed in the study by either patient or staff are listed in Supplementary Table 2, the time taken to perform the task, the type of delivery and the people involved are also listed.

**Additional file 4.** Practical Notes and Considerations. A description of some practical details and considerations to be taken into account when implementating the TnT service in a real-world setting.

**Additional file 5: Supplementary Fig. 1.** One-day timeline for two clinical staff providing TnT service – used to estimate maximum capacity (scenario 3). A timeline of the day for two healthcare staff, one student facing and one laboratory technician, representing the maximum number of tasks which they can perform when using three machines.

**Additional file 6: Supplementary Fig. 2.** One-day timeline for three 4-unit diagnostic machines – used to estimate maximum capacity (scenario 3). A timeline of the day representing the maximum number of students (47) that could be tested across 12 modules (3 machines). Students occupied one spot until all machines were saturated.

**Additional file 7: Supplementary Table 3.** Activities involved in the delivery of point of care testing and treating of Chlamydia, time taken, worker occupation and condition under which the process is required. A description of the processes involved in the point of care delivery is found in Supplementary Table 3, as well as the time required, the person actively performing the task and any conditions that might be associated with the task are also included.

## Data Availability

The datasets used and/or analysed during the current study are available on reasonable request from PO, the TnT trial’s Principal Investigator, via the corresponding author.

## Abbreviations

CT: Chlamydia trachomatis
HA: Health advisor
ISHT: Integrated Sexual Health Tariff
NCSP: National Chlamydia Screening Programme
NG: Neisseria gonorrhoea
POC: Point of care
RfPB: Research for Patient Benefit Programme
STI: Sexually transmitted infections
TnT: Test n Treat
