# Supplementary material for: Near patient chlamydia and gonorrhoea screening and treatment in further education/technical colleges: a cost analysis of the ‘Test n Treat’ feasibility trial
Source: BMC Health Serv Res. 2020 Apr 16;20:316. doi: 10.1186/s12913-020-5062-5 (PMC7160983; doi:10.1186/s12913-020-5062-5)
Supplement: Supplementary file 1 — Additional file 1: Supplementary Table 1. Costs and consumables required to deliver the Test n Treat service. The costs incurred throughout the study are listed in Supplementary Table 1, along with a description of the cost, purchase unit, quantity, type of cost and source of information are also included. [file 12913_2020_5062_MOESM1_ESM.docx]

**Supplementary Table 1. Costs and consumables required to deliver the Test ‘n’ Treat service**

| **Type** | **Description** | **Cost (£)** | **Unit** | **Quant-ity** | **Cost per day/**  **person** | **Source** |
| --- | --- | --- | --- | --- | --- | --- |
| Staff | Healthcare Assistant | £ 256.00 | Day | 200% | Day | 1 |
| Staff | Health Advisor | £ 416.00 | Day | 50% | Day | 1 |
| Staff | Travel | £ 11.20 | Day | 300% | Day | 1 |
| Services | Courier | £ 110.40 | Day | 100% | Day | 2 |
| Consumable | Apron | £ 0.16 | Item | 200% | Day | 3 |
| Consumable | BioCleanse | £ 0.63 | Vial | 2% | Day | 3 |
| Consumable | Clinical waste bags | £ 0.12 | Item | 200% | Day | 4 |
| Consumable | Sharps bin | £ 4.77 | Item | 100% | Day | 4 |
| Consumable | Testing machine | £ 184.20 | Item | 100% | Day | 5 |
| Consumable | Tablecloths | £ 1.89 | Item | 100% | Day | 6 |
| Consumable | Gloves | £ 0.28 | Pair | 200% | Day | 3 |
| Consumable | Label | £ 0.13 | Strip | 100% | Person | 7 |
| Consumable | Consent and info form | £ 0.10 | Item | 100% | Person | 4 |
| Consumable | Sample bag | £ 0.11 | Item | 100% | Person | 3 |
| Consumable | Urine pot | £ 0.10 | Item | 49% | Person | 3 |
| Consumable | Swab and buffer | £ 1.64 | Item | 51% | Person | 5 |
| Consumable | Pipette and buffer for urine | £ 1.64 | Item | 49% | Person | 5 |
| Consumable | Gloves | £ 0.28 | Pair | 103% | Person | 3 |
| Consumable | CT/NG test | £ 21.73 | Item | 103% | Person | 5 |
| Consumable | CT/NG test (repeat) | £ 21.73 | Item | 2% | Person | 5 |
| Consumable | Results text message | £ 0.05 | Text | 94% | Person | 8 |
| Consumable | Partner notification | £ 0.21 | Phone call | 0% | Person | 8 |
| Drugs | Azithromycin | £ 2.00 | Dose | 6% | Person | 9 |
| Consumable | Leaflets | £ 0.10 | Item | 100% | Person | 4 |
| Services | Waste disposal | £ 513.00 | Per tonne | 0.01% | Person | 4 |
| Services | Waste disposal | £ 513.00 | Per tonne | 0.12% | Day | 4 |

‘Per Day’ cost refers to a fixed daily cost irrespective of the number of screens performed. ‘Per person’ cost refers to a cost per person screened.

^1^ Personal Social Services Research Unit. Unit costs of Health and Social Care 2017. Available online [www.pssru.ac.uk/publications/pub-5380/](http://www.pssru.ac.uk/publications/pub-5380/) (Last accessed 13/03/2019)

^2^ Personal correspondence with Precision Cargo.

^3^ Fisher Sci.co.uk

^4^ SGUL internal procurement, published with permission and correct at the time of writing.

^5^ Cepheid, personal correspondence, based on monthly rental of 3 machines with 4 modules.

^6^ Sold by Unique Party on Amazon.co.uk

^7^ USA Scientific, Cryo-Babies.

^8^ Based on Pay as you go tariff on Three network.

^9^ British National Formulary. Available online <https://www.medicinescomplete.com/mc/bnf/current/PHP3502-azithromycin.htm> (Last accessed 13/03/2019)
