# Supplementary material for: Near patient chlamydia and gonorrhoea screening and treatment in further education/technical colleges: a cost analysis of the ‘Test n Treat’ feasibility trial
Source: BMC Health Serv Res. 2020 Apr 16;20:316. doi: 10.1186/s12913-020-5062-5 (PMC7160983; doi:10.1186/s12913-020-5062-5)
Supplement: Supplementary file 2 — Additional file 2. Calculating the maximum capacity of the TnT service (Scenario 3) [file 12913_2020_5062_MOESM2_ESM.docx]

### **Calculating the maximum capacity of the TnT service (Scenario 3)**

To calculate how many students could use the service if it was run at full capacity using three 4-unit machines per college (scenario 3), a simulation model was created in Python (Python Software Foundation. Python Language Reference, Version 2.7).(1) Each task was detailed including: the nature of the task (administrative or laboratory); its duration; whether it involved the student, the TnT team or both; and whether the task was associated with research, ‘real-world’ implementation or both – since some additional research-related resources and activities were required, such as obtaining consent and questionnaire completion, which would not happen if a similar service were rolled out (Supplementary Table 2, Additional file 3). A short summary of practical considerations and key learnings is included in the supplementary material (Additional file 4).

The maximum number of students who could be tested in one day was modelled using field data and the parameters listed in the next section. The model simulated the timelines of two healthcare staff: one student facing and one laboratory technician using three machines which could simultaneously test a total of 12 samples (Supplementary Figure 1, Additional file 5). Each student was added iteratively to the model, taking up respective space in the staff and machine timelines (Supplementary Figure 2, Additional file 6), until the timelines are saturated, and no more students could be added. Each student was simulated probabilistically and assigned characteristics based on the non-incentivised pathway parameters, forming an individual timeline. The healthcare worker who provided treatment for students with infections was not included in the model as this role tends to be more flexible and it is unlikely this person would have a saturated timeline. Data from one simulation were used to produce Gantt charts of the participant, healthcare worker and machine timelines for one day. The model was run for 1000 simulations.
