# Supplementary material for: Near patient chlamydia and gonorrhoea screening and treatment in further education/technical colleges: a cost analysis of the ‘Test n Treat’ feasibility trial
Source: BMC Health Serv Res. 2020 Apr 16;20:316. doi: 10.1186/s12913-020-5062-5 (PMC7160983; doi:10.1186/s12913-020-5062-5)
Supplement: Supplementary file 3 — Additional file 3 Supplementary Table 2. Processes required to deliver Test n Treat as research and as a service. All the procedures performed in the study by either patient or staff are listed in Supplementary Table 2, the time taken to perform the task, the type of delivery and the people involved are also listed. [file 12913_2020_5062_MOESM3_ESM.docx]

**Supplementary Table 2: Processes required to deliver Test n Treat as research and as a service**

| **Process** | **Time Taken** | | **Type of delivery** | | **People involved** | | **Source** |
| --- | --- | --- | --- | --- | --- | --- | --- |
|  | **Minutes** | **Seconds** | **Research** | **Implementation** | **Staff** | **Patient** |  |
| Explain TnT | 1 |  | ✓ | 🗶 | ✓ | ✓ | Estimate |
| Explain TnT | 2 |  | 🗶 | ✓ | ✓ | ✓ | Estimate |
| Record contact information | 3 |  | 🗶 | ✓ | ✓ | ✓ | Estimate |
| Look up name of participant, check phone number, DOB and ID | 1 |  | ✓ | 🗶 | ✓ | ✓ | Estimate |
| Unlock tablet and retrieve questionnaire | 0 | 23 | ✓ | 🗶 | ✓ | 🗶 | Replicated n=10 |
| Complete the questionnaire | 4 | 42 | ✓ | 🗶 | ✓ | 🗶 | From tablet logs n=91 |
| Label collection kit and put spare labels in pathology bag (Females) | 0 | 50 | ✓ | ✓ | ✓ | ✓ | Replicated n=10 |
| Label collection kit and put spare labels in pathology bag (Males) | 0 | 32 | ✓ | ✓ | ✓ | ✓ | Replicated n=10 |
| Go to bathroom and take sample (male) | 3 |  | ✓ | ✓ | 🗶 | ✓ | Estimate |
| Go to bathroom and take sample and put in buffer (female) | 4 |  | ✓ | ✓ | 🗶 | ✓ | Estimate |
| Wash hands & return | 1 | 30 | ✓ | ✓ | 🗶 | ✓ | Estimate |
| Record time on bag | 0 | 11 | ✓ | ✓ | ✓ | 🗶 | Replicated n=10 |
| Take bag to lab area | 0 | 10 | ✓ | ✓ | ✓ | 🗶 | Estimate |
| Staff member wears apron and gloves | 0 | 32 | ✓ | ✓ | ✓ | 🗶 | Replicated |
| Get new cartridge, buffer*, open sample bag | 0 | 20 | ✓ | ✓ | ✓ | 🗶 | Replicated once |
| Label lab sheet, cartridge, buffer with sample ID. Record time on sample bag. | 0 | 35 | ✓ | ✓ | ✓ | 🗶 | Replicated once |
| If urine: Add urine to buffer. Invert. | 1 | 0 | ✓ | ✓ | ✓ | 🗶 | Estimate |
| If swab: Add swab to buffer. Invert. | 0 | 10 | ✓ | ✓ | ✓ | 🗶 | Replicated once |
| Open cartridge. Transfer 1ml from buffer to cartridge. Replace buffer in store. | 0 | 55 | ✓ | ✓ | ✓ | 🗶 | Replicated once |
| Remove one glove | 0 | 4 | ✓ | ✓ | ✓ | 🗶 | Replicated n=20 |
| Select module and start test. Enter ID. Record time in buffer and module code. | 0 | 20 | ✓ | ✓ | ✓ | 🗶 | Estimate |
| Open and place the cartridge in the module. | 0 | 10 | ✓ | ✓ | ✓ | 🗶 | Estimate |
| Scan the barcode on the cartridge, and click “begin test” on computer | 0 | 5 | ✓ | ✓ | ✓ | 🗶 | Estimate |
| Once the light above the module flashes, close the module door to start the test. | 0 | 3 | ✓ | ✓ | ✓ | 🗶 | Estimate |
| Record the start time of the test (this is also recorded in the module) | 0 | 3 | ✓ | ✓ | ✓ | 🗶 | Estimate |
| Clean surface | 0 | 10 | ✓ | ✓ | ✓ | 🗶 | Estimate |
| Remove second glove | 0 | 4 | ✓ | ✓ | ✓ | 🗶 | Replicated n=20 |
| Machine runs test | 90 | 0 | ✓ | ✓ | ✓ | 🗶 | Cepheid |
| Read result, record on lab and results sheet | 0 | 45 | ✓ | ✓ | ✓ | 🗶 | Estimate |
| Put on gloves. Remove cartridge from module and discard. | 0 | 30 | ✓ | ✓ | ✓ | 🗶 | Estimate, glove time has been replicated |
| If the test failed, rerun steps from inversion of buffer tube. | 92 | 0 | ✓ | ✓ | ✓ | 🗶 | Estimate |
| Send negative result via text message | 0 | 45 | ✓ | ✓ | ✓ | 🗶 | Estimate |
| Pass positive result to health advisor to contact positive participants | 2 | 0 | ✓ | ✓ | ✓ | 🗶 | Estimate |
| Setting up: taking machines to room | 0 | 20 | ✓ | ✓ | ✓ | 🗶 | Estimate |
| Setting up: move tables, put down plastic, plug in and connect machines | 0 | 10 | ✓ | ✓ | ✓ | 🗶 | Estimate |
| Set up clinical waste bins | 0 | 30 | ✓ | ✓ | ✓ | 🗶 | Estimate |
| Taking down: Clean the modules and all surfaces | 1 | 0 | ✓ | ✓ | ✓ | 🗶 | Estimate |
| Taking down: Turn off computer and unplug machines | 5 | 0 | ✓ | ✓ | ✓ | 🗶 | Estimate |
| Taking down: pack up machines and take to exit | 20 | 0 | ✓ | ✓ | ✓ | 🗶 | Estimate |

Footnote: Where an average time is reported, n refers to the number of times this activity was timed in order to calculate the average.
