# Supplementary material for: Near patient chlamydia and gonorrhoea screening and treatment in further education/technical colleges: a cost analysis of the ‘Test n Treat’ feasibility trial
Source: BMC Health Serv Res. 2020 Apr 16;20:316. doi: 10.1186/s12913-020-5062-5 (PMC7160983; doi:10.1186/s12913-020-5062-5)
Supplement: Supplementary file 4 — Additional file 4. Practical Notes and Considerations. A description of some practical details and considerations to be taken into account when implementating the TnT service in a real-world setting. [file 12913_2020_5062_MOESM4_ESM.docx]

### Practical notes and considerations

This model requires a minimum of two people to run a consistent service; if one person is in the lab they cannot interact with the students at the same time. A sole worker could not run the student facing side of the service for two hours and then run the lab side; urine samples degrade at room temperature and should be transferred to the buffer within one hour, thus a one-person service would have a complex series of “opening hours” to meet this requirement, as well as increasing result turnaround.

“Real life” testing involved testing next day/overnight due to bulges in demand and failed test runs, so the “Test n Treat” model does not always fit neatly into a day of testing.

In the healthcare setting, if a clinic is not busy, staff can complete other tasks. In a community setting, this is not the case, so it is an inefficient use of staff time if the service is underused, so devices to increase uptake such as using incentives may be cost effective. Whilst high rates were detected in the incentivised follow up group, there is a risk that this incentive and the baseline incentive may have altered attitudes towards testing in college in general, however this may be balanced by the mitigation of stigma afforded by and external reason for testing. As treatment for gonorrhoea involves further testing and partner notification can take time not afforded by this approach, this form of community testing cannot be seen as a replacement for sexual health services in a medical setting.

The simulation was an approximation and did not consider students attending in groups, which may cut down time taken for steps “explain TnT” and “transfer sample”, however a slightly longer time would have been taken in providing the samples so this unlikely to affect the total number of samples predicted.

The cost data is built on the assumption that a site would be visited for one day and then a new site would be visited the next day. If the machines are to be used more than 20 times in a month, then costs would go down, less than this and the cost would go up. If the team visits a site for more than one consecutive day, then costs could go down as the cost of the courier and some daily costs such as for the sharps bin could be split over two days.
