# Supplementary figures and images for: Near patient chlamydia and gonorrhoea screening and treatment in further education/technical colleges: a cost analysis of the ‘Test n Treat’ feasibility trial
Source: BMC Health Serv Res. 2020 Apr 16;20:316. doi: 10.1186/s12913-020-5062-5 (PMC7160983; doi:10.1186/s12913-020-5062-5)

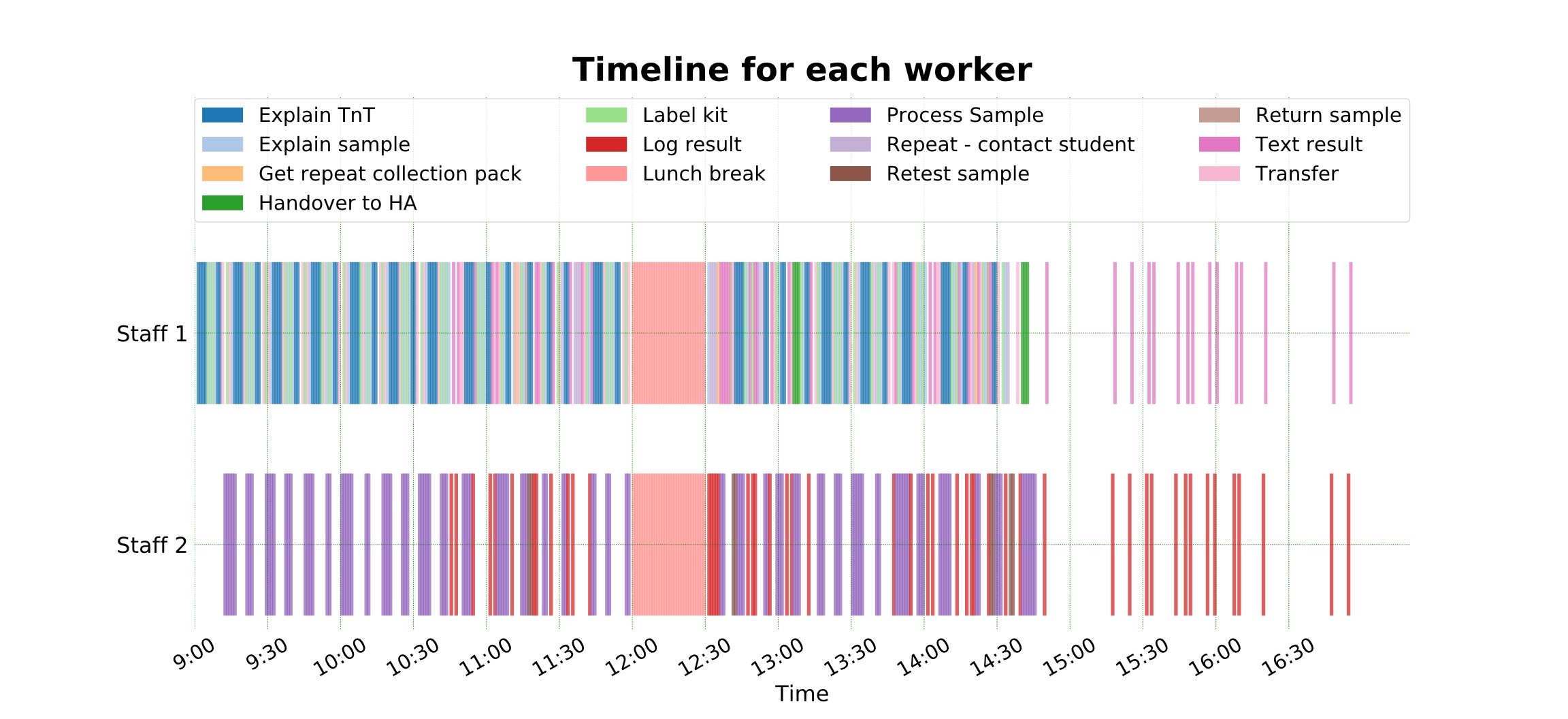

Supplement: Supplementary file 5 — Additional file 5: Supplementary Fig. 1. One-day timeline for two clinical staff providing TnT service – used to estimate maximum capacity (scenario 3). A timeline of the day for two healthcare staff, one student facing and one laboratory technician, representing the maximum number of tasks which they can perform when using three machines. [file 12913_2020_5062_MOESM5_ESM.jpg]

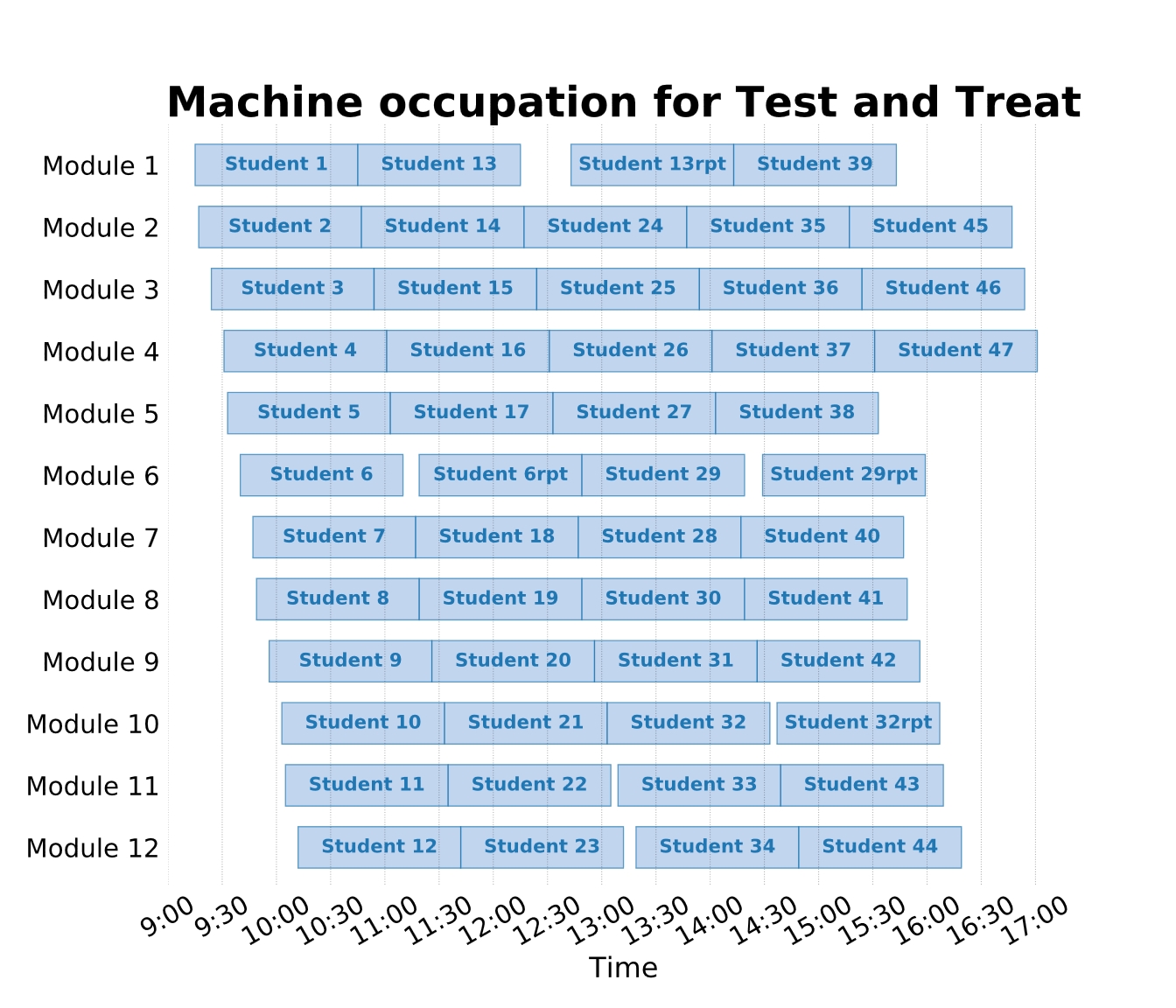

Supplement: Supplementary file 6 — Additional file 6: Supplementary Fig. 2. One-day timeline for three 4-unit diagnostic machines – used to estimate maximum capacity (scenario 3). A timeline of the day representing the maximum number of students (47) that could be tested across 12 modules (3 machines). Students occupied one spot until all machines were saturated. [file 12913_2020_5062_MOESM6_ESM.jpg]
