# Supplementary material for: Near patient chlamydia and gonorrhoea screening and treatment in further education/technical colleges: a cost analysis of the ‘Test n Treat’ feasibility trial
Source: BMC Health Serv Res. 2020 Apr 16;20:316. doi: 10.1186/s12913-020-5062-5 (PMC7160983; doi:10.1186/s12913-020-5062-5)
Supplement: Supplementary file 7 — Additional file 7: Supplementary Table 3. Activities involved in the delivery of point of care testing and treating of Chlamydia, time taken, worker occupation and condition under which the process is required. A description of the processes involved in the point of care delivery is found in Supplementary Table 3, as well as the time required, the person actively performing the task and any conditions that might be associated with the task are also included. [file 12913_2020_5062_MOESM7_ESM.docx]

**Supplementary Table 3: Processes involved in the delivery of point of care student testing and treatment of Chlamydia, time taken, worker occupation and condition under which the process is required**

| Process | Minutes | Student | Staff | Condition |
| --- | --- | --- | --- | --- |
| Explain TnT | 2 | Active | Admin |  |
| Record contact details | 3 | Active | Idle |  |
| Label collection kit | 1 | Idle | Active |  |
| Explain sample | 1 | Active | Admin |  |
| Student provides sample | 4 | Active | Idle | Male |
|  | 5 | Active | Idle | Female |
| Transfer sample | 1 | Active | Admin |  |
| Process sample | 3 | Idle | Lab | Male |
|  | 2 | Idle | Lab | Female |
| Run test | 90 | Idle | Idle |  |
| Record result | 1 | Idle | Lab |  |
| Resample contact | 3 | Active | Admin | Sample must be retaken |
| No resample contact | 1 | Active | Admin | Inform student sample must be retested |
| Get repeat collection pack | 1 | Active | Admin | Student will retake sample |
| Provide resample | 3 | Active | Idle | Male student will retake sample |
|  | 4 | Active | Idle | Female student will retake sample |
| Transfer sample | 1 | Idle | Admin | Student will retake sample |
| Retest sample | 2 | Idle | Lab | Student will retake sample OR retest with no resample |
| Rerun test | 90 | Idle | Idle | Student will retake sample OR retest with no resample |
| Record result | 1 | Idle | Lab | Student will retake sample OR retest with no resample |
| Handover to Health Advisor | 3 | Idle | Admin | Positive test result |
| Text result | 1 | Idle | Admin | Negative test result |
